# Artificial Intelligence, Deep Learning, and Computer Vision in Hysteroscopy: A Systematic Review

**DOI:** 10.3390/diagnostics16121899

**Published:** 2026-06-18

**Authors:** Rafał Watrowski, Attilio Di Spiezio Sardo, Peter Török, Andrea Rosati, Stoyan Kostov, Ibrahim Alkatout, Salvatore Giovanni Vitale

**Affiliations:** 1 Department of Gynecology, Helios Hospital Müllheim, Teaching Hospital of the University of Freiburg, Heliosweg 1, 79379 Müllheim, Germany; 2 Faculty of Medicine, University of Freiburg, 79106 Freiburg, Germany; 3 Department of Public Health, School of Medicine, University of Naples Federico II, 80138 Naples, Italy; attilio.dispieziosardo@unina.it; 4 Department of Obstetrics and Gynecology, Faculty of Medicine, University of Debrecen, 4032 Debrecen, Hungary; petertorokdr@gmail.com; 5 Department of Woman’s and Child Health and Public Health Sciences, Gynecologic Oncology Unit, Fondazione Policlinico Universitario Agostino Gemelli IRCCS, 00168 Rome, Italy; dott.andrearosati89@gmail.com; 6 Research Institute, Medical University Pleven, 5800 Pleven, Bulgaria; drstoqn.kostov@gmail.com; 7 Department of Gynecology, Hospital “Saint Anna”, 9002 Varna, Bulgaria; 8 Department of Obstetrics and Gynecology, Medical University of Innsbruck, 6020 Innsbruck, Austria; ibrahim.alkatout@i-med.ac.at; 9 Obstetrics and Gynecology Unit, “Gaspare Rodolico-San Marco” University Hospital, Department of General Surgery and Medical Surgical Specialties, University of Catania, 95124 Catania, Italy; salvatoreg.vitale@unict.it

**Keywords:** hysteroscopy, hysteroscopic, gynecology, artificial intelligence, deep learning, machine learning, computer-aided diagnosis, computer vision, neural networks, convolutional neural network

## Abstract

**Background/Objectives:** Hysteroscopy is the gold standard for visualization and treatment of intrauterine pathology. Because hysteroscopic interpretation remains operator-dependent, artificial intelligence (AI) has been evaluated as a tool to improve consistency, lesion recognition, and decision support. We aimed to systematically review AI, machine learning (ML), deep learning (DL), or computer-aided diagnosis (CAD) applications in hysteroscopy. **Methods:** A systematic search of PubMed/MEDLINE and EBSCOhost was performed from database inception to 8 March 2026, supplemented by targeted searches. Risk of bias was assessed using QUADAS-2 (diagnostic), PROBAST (prognostic), RoB2, and structured technical quality domains. **Results:** Nineteen primary studies were included, covering five areas: diagnostic classification and object detection (*n* = 8), real-time lesion detection and localization (*n* = 4), segmentation and visual-field support (*n* = 3), operative guidance (*n* = 1), and prognostic or decision-support applications (*n* = 3). Performance was highest in narrowly defined binary tasks and in large multicenter systems (e.g., ECCADx: AUC 0.979 internal, 0.975 external) and in prognostic fertility-prediction models after hysteroscopic adhesiolysis (AUC up to 0.992). Broader multiclass classification of heterogeneous lesions showed uneven and lower performance. Most studies were single-center, retrospective, and lacked external validation. Only one randomized study linked AI support to measurable procedural outcomes. **Conclusions:** The available studies indicate good technical performance in selected hysteroscopic tasks, particularly binary classification, focal lesion detection, and postoperative fertility stratification. Current evidence, however, remains limited by retrospective design, operator-dependent image acquisition, inconsistent validation, and scarce outcome-based clinical testing. In the short term, the most likely role of these systems is to support image interpretation, improve visual quality control, highlight suspicious lesions, and integrate hysteroscopic findings with complementary clinical data.

## 1. Introduction

Hysteroscopy remains the reference method for direct visualization and treatment of intrauterine pathology [1,2,3]. Improvements in optics and instrumentation have further expanded its use in outpatient settings, with high patient acceptability and low complication rates [4,5].

While other minimally invasive approaches, such as conventional, robotic-assisted, or NOTES laparoscopy, replaced or refined techniques that are, in principle, also feasible by open surgery, hysteroscopy developed into a distinct diagnostic and therapeutic field that cannot be replaced by alternative access routes. A targeted biopsy or removal of focal intrauterine pathology (polypectomy, submucosal myomectomy), the systematic intrauterine adhesiolysis, the correction of a septum, T-shape or niche—none of these are achievable by any other approach [6,7,8,9,10]. In selected early-stage cases of intrauterine malignancy, hysteroscopy enables uterus-preserving surgery under direct visualization—a therapeutic option that is not possible outside this access route [11,12].

These advantages are counterbalanced by substantial operator dependence. Macroscopic interpretation of mucosal color, vascular patterns, desquamation, protrusions, edema, fibrosis, and other subtle surface features depends heavily on the operator’s experience and on image quality [13,14,15,16]. In abnormal uterine bleeding, sensitivity for endometrial cancer or atypical hyperplasia increased from 55.5% in junior observers to 86.6% in experts, but inter-observer agreement remained only poor to fair [17,18]. Providing clinical information improves agreement, yet uncertainty persists for specific cavity abnormalities [19]. In addition, the sensitivity and specificity of hysteroscopy differ depending on type of pathology [14,20,21,22]. Structured training in hysteroscopy and standardized scoring systems are not uniformly implemented, and the learning curve remains steep, especially in low-volume centers [23]. These limitations highlight a need for tools that can standardize interpretation and support less-experienced operators.

**Figure 1:**
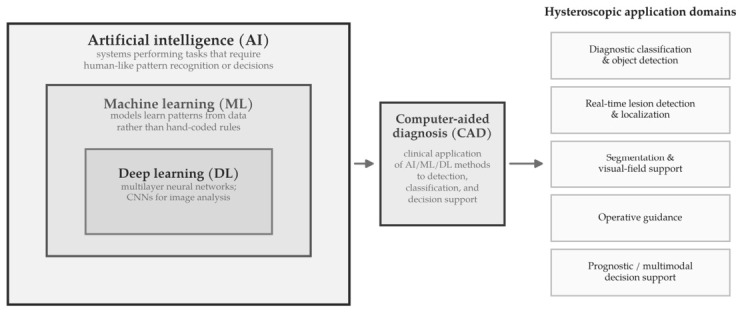
Conceptual taxonomy of the methods and applications reviewed. Artificial intelligence (AI), machine learning (ML), and deep learning (DL) form nested subsets. Convolutional neural networks (CNN) are the deep-learning architecture most relevant to hysteroscopic image analysis. Computer-aided diagnosis (CAD) denotes the clinical application of these methods to detection, classification, and decision support and is not a distinct algorithmic class.

Artificial intelligence (AI) is the broad umbrella term for computational systems designed to perform tasks that usually require human-like pattern recognition or decision-making. Machine learning (ML) is a subset of AI in which models learn patterns from data instead of manually coded rules. Deep learning (DL) is a further subset of ML based on multilayer neural networks, particularly effective for image analysis. Computer-aided diagnosis (CAD) refers to the clinical application of such methods to support detection, classification, or diagnostic decision-making, but is not itself a distinct algorithmic class [24,25,26,27]. The conceptual relationship between AI, ML, DL, and CAD, and the five hysteroscopic application domains used to organize this review, is shown in Figure 1. A glossary of the main AI and computer-vision terms used in this review is provided in Table A1.

AI, and particularly DL, has been increasingly applied across diagnostic medicine over the past decade. Convolutional neural networks (CNNs) have achieved high diagnostic performance in dermatology, ophthalmology, radiology and gastrointestinal endoscopy—tasks that share key features with hysteroscopic image analysis: pattern recognition in optical images, classification of texture and color anomalies, and detection of focal lesions against complex backgrounds [25,28]. The success of AI-assisted colonoscopy polyp detection—now supported by randomized controlled trials demonstrating a significant reduction in the adenoma miss rate [29]—provides a useful analogy for AI assistance in endoscopic procedures. These developments are directly relevant to hysteroscopy, where focal lesion detection, mucosal pattern analysis, and real-time visual interpretation are likewise central tasks.

Over the same period, AI applications have also expanded across benign and oncologic gynecology, particularly in imaging-rich domains [26,27,30]. The implementation of AI to integrate augmented reality and multimodal information (imaging techniques, radiomics, and molecular diagnostics) into a virtually enhanced surgical field led to a shift from “robotic-assisted” to “robotic-guided” laparoscopic surgery [31]. Existing reviews on AI in gynecology and gynecologic cancers summarize applications across ultrasound, MRI, CT, and colposcopy, and mention hysteroscopy only tangentially or as one of many imaging modalities [26,30]. Initial applications of AI, DL, and CAD in hysteroscopy suggest that AI may improve image interpretation, support lesion recognition, and assist selected intra- or post-procedural decisions [10,32,33,34]. To date, no focused systematic review has synthesized the small and heterogeneous evidence base on AI, ML, and DL specifically applied to hysteroscopic images, videos, and hysteroscopy-based prognostic models.

The objective of this systematic review is, therefore, to identify and assess studies that apply AI, ML, or DL to hysteroscopic imaging or hysteroscopy-based prediction tasks. We aim to (1) describe model architectures, input data, reference standards and validation methods; (2) summarize diagnostic performance for intrauterine pathology (benign, premalignant, and malignant) and prognostic performance for reproductive outcomes or post-treatment recurrence; and (3) explore how these systems compare with conventional hysteroscopic assessment and existing clinical workflows.

## 2. Methods

### 2.1. Study Design and Reporting Standard

This systematic review was conducted and reported in accordance with the “Preferred Reporting Items for Systematic Reviews and Meta-Analyses (PRISMA) 2020” guidelines [35], and the completed PRISMA checklist is provided in the Supplementary Materials. Owing to the methodological and clinical heterogeneity of the available literature, the review was designed as a qualitative evidence synthesis. The review protocol was not prospectively registered.

### 2.2. Eligibility Criteria

Studies were eligible if they met all of the following criteria: (1) original research, (2) clear relevance to hysteroscopy, (3) application of AI, ML, DL, or computer-aided diagnostic/decision-support systems, and (4) reporting of quantitative performance or outcome data.

We included studies in which AI was applied to hysteroscopic images, hysteroscopic videos, or hysteroscopy-relevant structured clinical/imaging data in one of the following domains:

- Diagnostic lesion classification or detection;
- Segmentation or visual-field support;
- Operative guidance;
- Postoperative prognostic prediction, multimodal prediction, or hysteroscopy-related decision support.

Studies were also eligible when hysteroscopy was a core clinical component of the AI application, even if the model additionally incorporated non-hysteroscopic variables or was designed to support hysteroscopy-related management decisions.

Conference abstracts without full methods and results were excluded. However, full-text conference/proceedings papers were eligible if they reported original data, fulfilled the predefined eligibility criteria, and were not superseded by a later, more comprehensive retained publication from the same research line or overlapping cohort.

Narrative reviews, editorials, commentaries, and non-original papers were excluded. When multiple publications from the same research group reported overlapping cohorts or iterative developmental stages of the same model, only the methodologically most complete and non-redundant report was retained in the main synthesis.

### 2.3. Information Sources and Search Strategy

The electronic literature search was performed in PubMed/MEDLINE and via the EBSCOhost platform, including Academic Search Premier, APA PsycArticles, APA PsycInfo, CINAHL, and MEDLINE, from database inception to 8 March 2026.

The PubMed search yielded 52 records using the following search string: “(hysteroscop*[Title/Abstract]) AND (“deep learning”[Title/Abstract] OR “machine learning”[Title/Abstract] OR “artificial intelligence”[Title/Abstract] OR “computer-aided diagnosis”[Title/Abstract] OR “computer aided diagnosis”[Title/Abstract] OR “convolutional neural network*”[Title/Abstract] OR “neural network*”[Title/Abstract] OR YOLO[Title/Abstract] OR FCNN[Title/Abstract] OR “support vector machine*”[Title/Abstract] OR transformer*[Title/Abstract])”.

The EBSCOhost search yielded 42 records using the following search string: “TX hysteroscop* AND TX (“artificial intelligence” OR “machine learning” OR “deep learning” OR “computer-aided diagnosis” OR “computer aided diagnosis” OR “computer-assisted diagnosis” OR “convolutional neural network*” OR “neural network*” OR YOLO OR “support vector machine*” OR transformer*)”.

In addition to database searching, a targeted supplementary search of Google Scholar and ResearchGate was performed to identify relevant full-text reports not indexed in the core databases. This supplementary search identified one additional full-text conference paper that met the eligibility criteria and is reported in the Results section.

### 2.4. Selection Process

**Figure 2:**
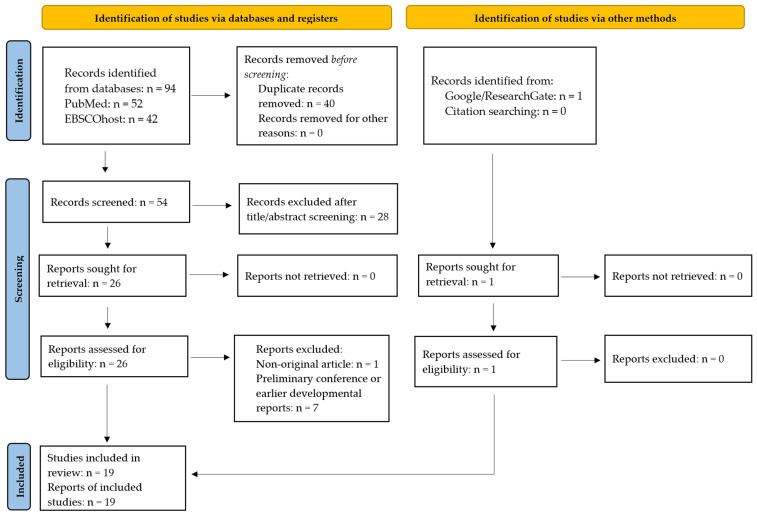
PRISMA 2020 flowchart of study identification and selection.

All records retrieved from PubMed and EBSCOhost were deduplicated before screening. Titles and abstracts were screened by two reviewers (R.W., S.K.), and potentially eligible reports underwent full-text assessment. Disagreements were resolved by discussion with other authors (A.R., S.G.V.). For studies published by the same research group on overlapping cohorts or representing successive developmental stages of the same model, a hierarchical inclusion strategy was applied: (1) preference for peer-reviewed journal articles over conference proceedings, (2) preference for publications with broader validation, especially external or multicenter validation, over single-center developmental reports, and (3) preference for the most recent and methodologically most complete publication when iterative model development was identified. No automation tools were used for study selection or data extraction. The selection process is visualized on the PRISMA 2020 flow diagram (Figure 2). After title/abstract screening, 27 full-text reports were assessed for eligibility. Finally, 19 primary studies were included in the final synthesis.

### 2.5. Data Extraction

Data were extracted into predefined evidence tables. The following items were collected for each study: full citation, country and center(s), clinical domain, study design, prospective versus retrospective design, number of patients, number of images/frames/videos, unit of analysis, clinical setting, lesion or target type, reference standard, AI model(s), comparator(s), validation approach, and quantitative performance or clinical outcome measures.

Where applicable, extracted performance metrics included the following: area under the curve (AUC/AUROC), sensitivity, specificity, accuracy, positive and negative predictive values, precision, recall, F1 score, Dice coefficient, intersection over union (IoU), mean average precision (mAP), concordance index (c-index), kappa statistics, and runtime or deployment-related information such as frames per second (FPS) or latency. For operative and prognostic studies, relevant clinical outcomes were also extracted, including operative time, blood loss, complete resection or incomplete resection, pregnancy-related outcomes, and treatment-benefit stratification.

### 2.6. Risk-of-Bias Assessment

Because the included literature comprised several different study types, risk of bias was assessed using study-type-specific instruments: QUADAS-2 [36] was used for diagnostic accuracy studies, PROBAST [37] was used for prognostic or predictive modeling studies, RoB 2 [38] was used for the randomized trial. For technical segmentation or visibility-related studies that did not fit a standard diagnostic-accuracy framework, we used a structured technical assessment of dataset selection, annotation quality, unit of analysis, data-leakage risk, validation strategy, and clinical relevance. Special attention was paid to AI-specific methodological issues, including selection bias, exclusion of poor-quality images, class imbalance, image-level instead of patient-level splitting, risk of overlap between training and test material, lack of external validation, unclear reference standards, overlap of datasets between publications, and limited reporting of calibration or deployment conditions.

### 2.7. Data Synthesis

Because of heterogeneity in target tasks, reference standards, model architectures, validation strategies, and reported metrics, statistical pooling was not considered appropriate. The studies were therefore synthesized narratively and organized into five thematic domains:

1. Diagnostic lesion classification and object detection;
2. Real-time lesion detection and localization;
3. Segmentation and visual-field support;
4. Operative guidance;
5. Prognostic, multimodal, and hysteroscopy-related decision-support applications.

Within these domains, results were interpreted in regard to study design, validation rigor, reference standard, and independence of evidence.

## 3. Results

### 3.1. Study Selection

The database search yielded 94 records, including 52 from PubMed and 42 from EBSCOhost. In addition, one further full-text report was identified through supplementary searching via Google/ResearchGate [39]. After removal of duplicates and title/abstract screening, 27 full-text reports were assessed for eligibility. Of these, eight reports were excluded after full-text review, leaving nineteen primary studies for inclusion in the final qualitative synthesis [39,40,41,42,43,44,45,46,47,48,49,50,51,52,53,54,55,56,57].

Full-text exclusions comprised one narrative review/non-original article [33], two earlier developmental reports from the intrauterine adhesion prediction line [58,59], two preliminary conference/developmental reports from the CATIA line [60,61], one prototype/integrated developmental report from the same CATIA research line [62], and two earlier developmental or conference-stage reports from research lines later represented by more comprehensive retained studies [63,64]. The final dataset therefore consisted of 19 primary studies, including both peer-reviewed journal articles and eligible full-text conference/proceedings papers.

### 3.2. Study Characteristics

Study characteristics are summarized in Table 1, and the study-quality/risk-of-bias overview is given in Table A2. The evidence from 19 included studies covering diagnostic lesion classification and detection, segmentation and visual-field support, operative guidance, and prognostic or decision-support applications. Most studies were retrospective or retrospective in silico; one study used a randomized prospective comparison of AI-assisted operative planning, and the fertility-prediction studies from the Beijing group were based on prospectively assembled intrauterine adhesion cohorts [50,52,56]. Dataset size varied markedly, from 52 subjects and 516 ROIs in the CAD study by Neofytou et al. [41] to 1394 patients and 55,874 hysteroscopy images in the multicenter ECCADx study by Wang et al. [54]. The technically oriented segmentation studies were generally based on comparatively small datasets. For example, Wang et al. [47] analyzed 1385 hysteroscopic images for bubble segmentation, whereas Burai et al. [43] used 28 hysteroscopic videos for uterine-wall segmentation.

The included studies did not rely on a single reference standard, reflecting their different clinical and technical aims. Histopathology was used in several lesion-classification studies [40,41,44,53,55]; Kitaya et al. evaluated expert-annotated EMiP recognition within a histologically confirmed CE cohort [51]. Expert annotation served as the reference for object-detection and segmentation studies, including fibroid, polyp, uterine-wall, and bubble detection or segmentation [42,43,46,47,49]. In the prognostic studies by Li et al., reproductive follow-up after hysteroscopic adhesiolysis was used as the outcome reference [52,56]. In Givon et al. [57], incomplete hysteroscopic myomectomy was defined as residual submucosal myoma documented at the end of surgery.

Across the nineteen studies, nine were judged at high or high/unclear risk of bias—including all three prognostic models assessed with PROBAST—and eight at moderate to moderate–high risk, while the single randomized trial carried “some concerns” (RoB 2); the recurring sources were absent of external validation, had single-center retrospective design, and used image- or frame-level rather than patient-level data splitting (Table A2).

### 3.3. Lesion Classification and Object Detection

Diagnostic classification and object detection studies are summarized in Table 2. Early work relied on handcrafted descriptors and conventional classifiers. Vlachokosta et al. classified three categories of hysteroscopic images, derived from 28 patients with abnormal uterine bleeding, 10 with endometrial cancer, and 39 normal subjects, and reported an accuracy of 91.2%, sensitivity of 93.6%, and specificity of 83.8% using an artificial neural network after reduction of 167 vessel and texture features to four selected features [40]. Neofytou et al. developed a CAD system for early endometrial cancer detection based on gamma-corrected red–green–blue (RGB), hue–saturation–value (HSV), and YCrCb color-space texture analysis in 516 regions of interest (ROIs) from 52 subjects; the best ROI-level result was an 81% correct classification rate with statistical features plus gray-level difference statistics (SF + GLDS) and a support vector machine (SVM) [41].

Later deep-learning studies expanded both lesion spectrum and dataset size. Takahashi et al. analyzed 177 patients with normal endometrium, myoma, polyp, atypical endometrial hyperplasia, or endometrial cancer and showed that diagnostic accuracy increased to 90.29% after continuity analysis and model combination; sensitivity and specificity were 91.66% and 89.36%, respectively [45]. Zhang et al. used 1851 histologically confirmed images from 454 patients and reported an overall five-class accuracy of 80.8% for endometrial hyperplasia without atypia, atypical hyperplasia, endometrial cancer, endometrial polyp, and submucous myoma. In the same study, binary classification of benign versus premalignant or malignant lesions performed better, with an accuracy of 90.8%, sensitivity of 83.0%, and specificity of 96.0%; class-specific AUCs ranged from 0.916 for atypical hyperplasia to 0.981 for endometrial polyp [44].

A similar pattern was seen in the multiclass work by Raimondo et al. [53]. In 266 patients contributing 1500 images, lesions were grouped as benign focal, benign diffuse, and preneoplastic or neoplastic. The best classification performance reached an overall accuracy of 86.74%, specificity of 90.06%, and an F1 score (harmonic mean of precision and recall) of 80.11%. However, class-wise performance was uneven. In the best-performing configuration, recall was 94.07% for benign focal lesions, 50.0% for benign diffuse lesions, and 27.59% for preneoplastic or neoplastic lesions. In the separate identification task, the best model, obtained with clinical factors, achieved detection of 85.82%, precision of 93.12%, recall of 91.63%, and F1 score of 92.37%.

Several studies focused on binary or narrower target recognition and generally reported higher performance. Kitaya et al. analyzed fluid hysteroscopic images from 208 infertile women and developed a CNN for automatic detection of endometrial micropolyps associated with chronic endometritis (CE). Sensitivity, specificity, accuracy, precision, and F1 score were 93.6%, 92.3%, 92.8%, 88.0%, and 0.907, respectively, and the AUC was 0.930. The model’s AUC was statistically comparable to that of three experienced gynecologists, whose AUCs ranged from 0.906 to 0.948 [51].

The largest and methodologically strongest diagnostic study was the multicohort ECCADx system by Wang et al. [54]. In the final report, 1394 patients contributed 55,874 hysteroscopy images, with 1204 patients in the training set, 85 in the internal MCH test set, and 105 in the external TJH/ZZSH test set. With contrastive learning, internal test performance reached an AUC of 0.979, accuracy of 94.1%, sensitivity of 95.2%, specificity of 91.3%, and F1 score of 0.959. On the external test set, the corresponding values were AUC 0.975, accuracy 93.3%, sensitivity 92.1%, specificity 100%, and F1 score 0.959. Without contrastive learning, external specificity was 62.5%, showing a marked gain after domain-adaptive training. The authors also compared ECCADx with junior, medium, and senior gynecological endoscopists and found that the model outperformed human readers on both internal and external datasets [54]. A smaller report by Kürkçü et al. [39] evaluated four YOLO variants on 1482 physician-annotated hysteroscopy images of polyps, myomas, and endometrial cancer. The prototype was intended to support clinicians by localizing suspicious hysteroscopic abnormalities for review. YOLOv9c achieved the highest mAP50, defined at an intersection-over-union (IoU) threshold of 0.50, with 0.906, and the highest precision with 0.894, whereas YOLOv8s achieved the highest recall with 0.906; expert review of 50 images yielded model accuracies of 94–98%.

Across these studies, narrowly defined binary or lesion-specific tasks generally yielded stronger and more stable performance than broader multiclass differential diagnosis. This was shown most explicitly in the five-class versus binary comparison by Zhang et al. [44] and was also reflected by the class imbalance and low minority-class recall in the three-category study by Raimondo et al. [53]. By contrast, lesion-specific systems for micropolyps or AEH/endometrial cancer generally reported higher sensitivity, AUC, or F1 values [51,54].

### 3.4. Real-Time Lesion Detection and Localization

Real-time lesion detection and localization studies are summarized in Table 3. Several studies focused on object detection or real-time lesion recognition in hysteroscopic images and videos. A CNN-transformer hybrid system developed for uterine fibroid recognition achieved in the test set of 2312 images sensitivity 94.21%, specificity 83.76%, accuracy 88.93%, F1 score 89.36%, and AUC 0.96 [48].

A modified YOLOX model was trained on 11,839 images from 323 polyp cases and evaluated on internal and external hospital datasets comprising 431 cases in total. Lesion-based sensitivity reached 100% on the internal test set and 92.0% on the external test set, compared with 95.83% and 77.33%, respectively, for the original YOLOX model [49]. The proposed system processed video at 63 frames per second. In an earlier conference study, the same group reported real-time hysteromyoma detection with a YOLOv3 plus deep convolutional generative adversarial network (DCGAN) hybrid model at 25 frames per second (FPS) and an accuracy of 91.73% [46].

Mascarenhas et al. trained a YOLOv1-based multicentric model on 65 hysteroscopies yielding 33,239 frames and 37,512 annotated objects. On the test set, object-level recall was 0.96, precision 0.95, mAP50 was 0.98, and mAP50-95 was 0.77. At frame level, mean recall was 0.75, mean precision 0.98, and mean F1 score was 0.82 [55].

In sum, these studies indicate that real-time lesion detection and localization are feasible in hysteroscopy, particularly for visually distinctive focal pathology such as fibroids and polyps, although validation remains limited in most datasets.

### 3.5. Segmentation and Visual-Field Assessment

Segmentation and visual-field support studies are summarized in Table 4. Török and Harangi [42] analyzed 13 videos of hysteroscopic myomectomy and trained a fully convolutional neural network (FCNN) on 4688 annotated images, with 1600 previously unseen images reserved for testing. The model aimed to identify the plane between myoma and normal myometrium and achieved a reported pixel-wise segmentation accuracy of 86.19%. Burai et al. addressed uterine-wall extraction in hysteroscopic video frames affected by bubbles, instruments, and detached mucosa. Their ensemble of FCNNs yielded a best Dice coefficient of 0.9156 and Jaccard index of 0.8443 [43]. Wang et al. [47] focused on bubble segmentation during operative hysteroscopy, using 1385 clear images. Their edge-aware network achieved accuracy of 0.859 ± 0.017, sensitivity of 0.868 ± 0.019, precision of 0.955 ± 0.005, Dice score of 0.862 ± 0.005, and mean intersection over union of 0.758 ± 0.007. Compared with U-Net, Dice improved from 0.819 to 0.862 and mean IoU from 0.713 to 0.758. Reported inference time was 0.15 s per image.

These segmentation studies addressed different technical problems and are therefore not directly comparable, but they show that hysteroscopy-oriented AI has extended beyond diagnosis toward intraoperative scene parsing, field stabilization, and extraction of visually relevant structures [42,43,47].

### 3.6. Operative Planning and Guidance

The only included operative-guidance study is summarized in Table 5. Chen et al. [50] randomized 56 patients with submucosal myomas to standard MRI-based preoperative planning or AI-augmented MRI-based planning before hysteroscopic myomectomy. The AI-assisted group had a shorter operative time than the control group (32.11 ± 11.86 vs. 41.32 ± 17.83 min, *p* = 0.03) and a (clinically likely irrelevant) lower intraoperative blood loss (median 10.00 [5.00–15.00] vs. 10.00 [6.25–15.00] mL, *p* = 0.04). This was the only included study that directly linked AI support to a measurable downstream procedural effect, although the AI input in this study was MRI segmentation rather than real-time hysteroscopic image analysis.

### 3.7. Prognostic and Preoperative Decision Support

Prognostic, multimodal, and preoperative decision-support studies are summarized in Table 6. Two studies came from the Beijing intrauterine adhesion database. Li et al. [52] developed a multimodal learning system that integrated EMR variables with 5014 revisited hysteroscopic images from 753 post-adhesiolysis patients. Using MobileNetV3 for image feature extraction and XGBoost for multimodal ensemble learning, the model achieved AUCs of 0.967 in training, 0.936 in validation, and 0.965 in the test set for one-year conception prediction. The system operated on the hysteroscopic platform with an average analysis time of 3.7 ± 0.8 s per patient. Risk stratification based on predicted conception probability was clinically informative: mid-high-risk patients had significant benefit from assisted reproductive technology, with an odds ratio of 6 (95% CI 1.27–27.8; *p* = 0.02), whereas low-risk patients showed no significant ART benefit.

In a related image–deep-learning study [56], Li et al. analyzed 555 cases with 4922 hysteroscopic images and developed a proportional-hazard CNN system for fertility assessment after endometrial injury. Reported AUCs were 0.982, 0.992, and 0.990 across the three randomly assigned datasets in the abstract; after excluding patients who underwent ART within one year, the corresponding AUCs were 0.982, 0.992, and 0.989. Net benefit at the chosen intervention threshold reached 69.4% in the test cohort. Concordance indices for the proportional-hazard CNN were 0.940, 0.920, and 0.925 in the training, validation, and test cohorts, respectively. Agreement between the system’s quantified risk-factor panel and senior hysteroscopist assessment was high, with kappa values ranging from 0.84 to 0.89 across four intrauterine pathological domains.

Givon et al. [57] evaluated a different use case: preoperative prediction of incomplete hysteroscopic myomectomy using routine clinical variables together with ultrasound and diagnostic hysteroscopy findings. In 345 procedures from 328 women, incomplete resection occurred in 16.2%. CatBoost, trained with stratified five-fold patient-level cross-validation, achieved an AUROC of 0.72 and average precision of 0.93; at the prespecified 0.50 threshold, accuracy was 0.76, positive predictive value 0.89, sensitivity 0.81, specificity 0.50, and F1 score 0.85. In a fixed preprocessed comparator analysis, CatBoost reached accuracy 0.69, positive predictive value 0.88, sensitivity 0.73, specificity 0.50, F1 score 0.80, AUROC 0.71, and average precision 0.93, modestly outperforming logistic regression trained on identical inputs.

### 3.8. Performance by Task Type

Across domains, the strongest performance was reported in tightly defined tasks with clear target labels, including binary AEH/endometrial cancer discrimination, endometrial micropolyp detection, focal polyp or fibroid localization, and postoperative fertility prediction after adhesiolysis [51,54,55,56]. Broader multiclass classification of heterogeneous intrauterine lesions was less stable, and the lowest class-specific recall was reported for the preneoplastic/neoplastic category in the Raimondo study [53]. Segmentation and visual-field support studies showed technical feasibility, often with good Dice or IoU values, but did not report patient-level diagnostic or clinical endpoints. Chen et al. [50] was the only study that directly linked AI support to procedural outcomes, whereas Li et al. [52,56] directly linked hysteroscopy-based AI outputs to postoperative reproductive stratification.

## 4. Discussion

This review synthesized nineteen primary studies applying AI, ML, and DL to hysteroscopy across diagnostic, technical-support, operative, and prognostic tasks. Across these studies, performance is strongest in large binary diagnostic systems for AEH and endometrial cancer and in narrow prognostic models for postoperative fertility after adhesiolysis. The ECCADx system, achieving an AUC of 0.979 on internal validation and 0.975 on external multicenter data, is among the highest reported in AI-based gynecological diagnostics [54]. At the same time, the overall evidence base remains preliminary. Most studies were retrospective, most were single-center, and only one directly evaluated a clinical procedural endpoint in a comparative design [50]. The available evidence therefore establishes technical feasibility in selected tasks but does not yet demonstrate prospective clinical effectiveness or workflow superiority in routine practice, neither of which can be inferred from retrospective, single-center accuracy estimates.

A consistent pattern across the reviewed literature is the difference between performance in narrow, well-delimited tasks and performance in broader multiclass classification. In Zhang et al. [44], binary discrimination between benign and premalignant/malignant lesions outperformed five-class classification within the same dataset. In Raimondo et al. [53], overall accuracy remained acceptable (87%), but recall for the preneoplastic/neoplastic category fell to 28%, meaning that more than 70% of cases in this category were not detected as such by the model.

The best-supported hysteroscopic applications are image-based recognition tasks: lesion classification, focal lesion localization, and detection of subtle inflammatory patterns. These are also tasks in which human interpretation varies substantially with experience, image quality, and clinical context [14,16,18,19]. At the current stage, the most plausible role of AI is not “replacement” of the hysteroscopist, but support for more consistent recognition of patterns that may otherwise be overlooked, overcalled, or interpreted differently across operators. Chronic endometritis (CE) is a good example of a setting in which AI may be clinically useful despite only partially overlapping hysteroscopic, histopathological, immunohistochemical, and microbiologic reference standards [65]. The CNN for automatic detection of endometrial micropolyps associated with CE developed by Kitaya et al. [51] achieved an AUC of 0.930, comparable to experienced gynecologists.

Examples of AI narrowing the performance gap between junior and more experienced examiners are also available from gynecological ultrasonography. In the study by Xu et al., a YOLOv8-based model for deep infiltrating endometriosis improved the AUROC of two junior sonologists from 0.748 to 0.878 and from 0.713 to 0.798, with sensitivity increasing up to 94.35% [66]. This supports the broader point that AI may be particularly useful in operator-dependent visual diagnostics, where expert-level pattern recognition is difficult to acquire and immediate supervision is not always available. Deep learning has also been applied to 3D transvaginal ultrasound for intrauterine adhesions, reaching high accuracy with external validation [67]. For T-shaped uterus, marked discordance between competing 3D-ultrasound diagnostic criteria illustrates the instability of morphometric thresholds [68], while a recent machine-learning model based on quantitative 3D-TVUS parameters achieved good diagnostic discrimination [69]. At the same time, poor-to-moderate inter-observer agreement for a proposed gynecological TVS image-quality scoring system shows that variability begins already at image acquisition and labeling [70].

Hysteroscopic image quality depends heavily on the procedure itself. Distance from tissue, viewing angle, bleeding, specular reflections from the fluid-distension medium, variable cavity distension, motion blur, and the curved, partially occluded field of view all shape the input available to the model. This differs from colonoscopy and gastroscopy, where larger benchmark datasets and better-developed artifact frameworks have supported more generalizable systems [71,72,73,74]. In hysteroscopy, the studies by Wang et al. on bubble segmentation [47], by Burai et al. [43] on uterine-wall extraction, and the myomectomy plane segmentation by Török and Harangi [42] address technical conditions that are directly relevant for practical deployment.

Translational adoption also depends on clinically interpretable output and reliable uncertainty estimates at the point of care. The deep-learning models that perform best on hysteroscopic images are largely opaque, and the resulting “black-box” character, together with the need for interpretability, calibrated confidence, and demonstrable fairness, is now recognized as a principal barrier to clinical deployment of AI in medical imaging [24]. None of the included hysteroscopic studies reported calibration of predicted probabilities or provided interpretability output that a clinician could use to accept or override a given prediction. For real-time hysteroscopy, retrospective expert-level accuracy is insufficient unless the output is interpretable and its uncertainty is clearly communicated during the examination. Confidence calibration and interpretable localization are needed before these systems can support intraoperative decisions.

Hysteroscopy, at its current developmental stage, remains a manually performed procedure. Navigation, lesion exposure, tissue handling, instrument positioning, and the final operative decision still depend on the operator’s real-time judgment. This limits direct comparison with laparoscopy, especially robotic laparoscopy, where the operative interface is already digital and telemanipulated, preoperative imaging can be overlaid onto the operative field, critical structures can be annotated in real time, and workflow analysis can be integrated more directly into surgical guidance [27,75,76,77]. Hysteroscopy does not currently offer an equivalent platform. The main contribution of AI is more likely to lie in perception support, image-quality control, lesion highlighting, multimodal integration, and postoperative prediction.

The reviewed studies point to four near-term applications: (1) image processing and classification during or after the procedure, as a decision-support layer that augments interpretation without replacing manual control [53,54,55]; (2) texture and vascular-pattern analysis for subtle pathology, including chronic endometritis [51], early hyperplasia [44,54], and structural anomalies; (3) multimodal integration with 3D ultrasound or sonohysterography for pre-hysteroscopic planning in T-shaped uterus, uterine septa, and Asherman syndrome [52,59], where cavity-level and panoramic imaging provide complementary information; and (4) AI-assisted postoperative outcome prediction, as demonstrated by the Beijing group [52,56,57,59], in which hysteroscopic findings contribute to probabilistic clinical decision-support tools.

### 4.1. Limitations

Risk-of-bias assessments consistently show that technically sound model development is undermined by limited validation. This pattern is not specific to hysteroscopy, as several clinical studies evaluating AI in surgery rely on sparse multicenter data, heterogeneous annotation standards, limited external validation, and insufficient outcome-oriented evaluation [78]. Fourteen of the nineteen included studies were retrospective and single-center. Many used internal train–test splits from the same institutional archive. Under these conditions, performance may be inflated by shared acquisition settings, equipment, annotation habits, or patient mix [78,79,80,81,82]. This problem is compounded when the unit of analysis is the image or frame rather than the patient. If multiple images from the same patient are distributed across training and test sets, effective sample size is overstated and leakage may remain undetected.

A further constraint concerns the provenance of the datasets themselves. Most included studies drew on curated retrospective archives in which low-quality frames were excluded before model development, so that the images used for training and testing represent favorable acquisition conditions instead of routine hysteroscopic environment, in which glare, bleeding, mucus, debris, motion blur, and incomplete cavity distension are common. Annotation reference standards were heterogeneous and frequently rested on a single expert reader or on the interpreting clinician, without reported inter-annotator agreement, so that label noise cannot be quantified. This combination of curated inputs and single-source labels limits ecological validity, as reported performance reflects development conditions rather than real-world deployment.

The heterogeneity of reporting across studies makes cross-study comparison unreliable. Studies report different primary metrics (AUC, accuracy, F1, Dice, C-statistic), use different reference standards (histopathology from directed biopsy, expert hysteroscopic annotation, blinded panel review), and employ different units of analysis (per-image, per-frame, per-patient). The choice of per-image instead of per-patient analysis artificially inflates effective sample sizes and can mask patient-level confounding: when multiple images from the same patient are split across training and test sets—a form of data leakage whose performance-inflating effects have been quantified at 30–55% in CNN-based classification tasks [81,82]—reported performance metrics are optimistic in ways that are practically impossible to detect without access to the original data. For the detection and localization studies specifically, the unit of analysis, the intersection-over-union threshold defining a true detection, and whether negative frames or videos were included in the test set were reported inconsistently; this incomplete reporting limits comparison across studies. Inter-observer variation in histopathological diagnosis of endometrial hyperplasia remains substantial even among specialist pathologists [83], compounding the difficulty of constructing unambiguous reference standards for AI training. The study by Raimondo et al. [53] illustrates this limitation: overall classification accuracy of 86.74% coexisted with a recall of only 27.59% for the preneoplastic/neoplastic class, precisely the category of greatest clinical relevance and the one for which a missed diagnosis carries the highest patient risk.

The same limitation appears differently in the prognostic studies. Their reported AUCs are high, but validation remains internal to a single cohort line [52,56]. Calibration of predicted probabilities was rarely reported. Li et al. [56] included decision-curve analysis and reported a net benefit of 69.4% for the fertility-assessment system, whereas Li et al. [52] reported risk-stratified ART benefit without formal probability calibration. Earlier Cyprus CAD work provided an internally validated texture-based proof of concept [41]. The Beijing intrauterine-adhesion models report high AUCs, but validation remains internal to one cohort line [52,56]. Wang et al. [54] provide the strongest diagnostic validation with independent multicenter test datasets; Chen et al. [50] remains the only study with a comparative procedural endpoint. The imbalance between tasks where AI implementation produces statistically significant numbers and their clinical utility can be illustrated by two studies addressing hysteroscopic myomectomy [50,57]. The work of Chen et al. [50] remains the only randomized comparison linking AI support to a procedural endpoint. It reports a shorter operative time (around 10 min) and statistically lower blood loss despite identical median values in both groups and only slightly different interquartile ranges. The clinical relevance, however, remains speculative. First, the almost identical and extremely low blood loss contrasted with a difference of approximately 25% in surgical time. It is surprising, because surgical time is a strong predictor of blood loss at least in, e.g., laparoscopic myomectomy [84,85]. By contrast, Givon et al. [57] addressed the clinically relevant problem of incomplete myomectomy. In real-world settings, incomplete myomectomy has been reported in 36.69% of hysteroscopic approaches and is associated with complications (PR: 2.77; 95% CI: 1.43–5.38) [86]. The model performance in [57] was clearly lower than in the Beijing fertility studies or ECCADx, but the results suggest that AI can help identify cases in which complete hysteroscopic treatment is less likely.

### 4.2. Future Directions

Progress toward clinical use requires prospective multicenter validation. The primary outcomes should include, beyond image-level accuracy, patient-relevant measures, such as pregnancy, detection of (pre)malignant lesions, recurrence, or completeness of treatment. Where possible, analyses should also be stratified by operator experience, because support systems may be most useful for less-experienced hysteroscopists, as demonstrated by Xu et al. in the ultrasound setting [66]. Reporting standards for early-stage clinical evaluation of AI decision-support systems, as formalized in the DECIDE-AI guideline, should be adopted to prospective hysteroscopic AI studies [87].

The parallel development of standardized hysteroscopic image databases will be needed for generalizable models and cross-study comparison. Such databases require standardized image-acquisition protocols and agreed annotation standards for specific intrauterine pathologies. Coordinated international annotation initiatives, analogous to those that underpinned the CVC-ClinicDB and REAL-Colon colonoscopy benchmarks, are necessary for progress [71,72,73].

Multimodal AI, combining hysteroscopic images with 3D ultrasonography, MRI findings, hormonal profiles, and clinical variables, is a plausible next step. The success of the Beijing multimodal system [52] suggests that the discriminative power of hysteroscopic images is substantially amplified when contextualized by complementary data modalities. Extending this principle to diagnostic tasks, for example by integrating imaging studies (MRI, 2D- or 3D-TVS) or electronic medical records with real-time hysteroscopic assessment, could address the limits of image-only approaches [56,75].

Finally, AI-driven training simulators that provide real-time feedback on instrument handling and pattern recognition may help shorten the steep learning curve in hysteroscopy and reduce interobserver variability, particularly in settings with limited expert supervision [24,88].

These training and decision-support roles raise an ethical question that cannot be separated from the technical one. Pope Leo XIV’s first encyclical, *Magnifica Humanitas*, addresses the place of the human person in the age of artificial intelligence and warns that ready-made answers may “weaken personal creativity and judgment” and that easy retrieval can “risk extinguishing the desire to ask questions” [89]. Medical research expresses a related concern through co-intelligence, a model of human–AI collaboration that preserves clinician judgment and treats AI literacy as a core competence [90]; medical imaging literature similarly identifies interpretability and trust as prerequisites for adoption [24]. In hysteroscopy, AI should therefore be judged by diagnostic performance and by its effect on operator judgment, procedural skill, and the questioning that real-time intrauterine decision-making still requires [50,87].

## 5. Conclusions

The existing literature shows that machine-learning and deep-learning systems can classify endometrial lesions, detect focal pathology in real time, segment relevant intraoperative structures, and support selected prognostic decisions. The strongest evidence currently concerns binary diagnostic classification for AEH and endometrial cancer, focal lesion detection, and fertility-related risk stratification after hysteroscopic treatment of intrauterine adhesions. At the same time, the field remains constrained by retrospective design, operator-dependent image generation, limited external validation, heterogeneous reference standards, and scarce evaluation of patient-centered outcomes. Unlike laparoscopy, where AI-robotics integration is moving toward real-time guidance and image overlay, hysteroscopy remains a manually performed procedure. Near-term use is therefore likely to remain concentrated in image processing, texture analysis, multimodal data integration, and decision-support. These systems are most likely to support perception by improving interpretation consistency, highlighting suspicious lesions, assisting biopsy targeting, and integrating hysteroscopic findings with clinical data. Translating these technical achievements into clinical practice requires prospective multicenter validation, standardized datasets, and workflow integration.

## Figures and Tables

**Table 1:** Characteristics of the included primary studies.

Study	Domain	Dataset	Task	Key Result
Vlachokosta 2013 [40]	Diagnostic classification	77 women: 28 AUB, 10 EC, 39 without pathology	3-class classification: normal vs. AUB vs. EC	Accuracy 91.2%; sensitivity 93.6%; specificity 83.8%
Neofytou 2015 [41]	Diagnostic classification	516 ROIs from 52 subjects	Normal vs. abnormal endometrium/early EC-oriented CAD	Best correct classification rate 81% (SF + GLDS with SVM)
Török 2018 [42]	Operative/technical support	13 surgical cases; 4688 training images and 1600 test images	Segmentation of normal myometrium vs. myoma plane	Pixel-wise segmentation accuracy 86.19%
Burai 2018 [43]	Operative/technical support	28 videos; 1425 subimages	Uterine wall segmentation under occluding conditions	Segmentation accuracy 91.56%; Dice 0.9156; Jaccard 0.8443
Zhang 2021 [44]	Diagnostic classification	1851 images from 454 patients; test set 250 images	5-class lesion classification; benign vs. premalignant/malignant	Overall accuracy 80.8%; benign vs. premalignant/malignant: accuracy 90.8%, sensitivity 83.0%, specificity 96.0%
Takahashi 2021 [45]	Diagnostic classification	177 patients	EC-focused diagnosis from hysteroscopy	Combined accuracy 90.29%; sensitivity 91.66%; specificity 89.36%
Zhao 2021 [46]	Detection/localization	87 videos from 45 patients; additional test on 8 videos from 5 patients	Real-time hysteromyoma detection	Accuracy 91.73%; real-time speed 25 FPS
Wang 2022 [47]	Operative/technical support	12 videos from 12 patients; 1385 clear images	Bubble segmentation and size distribution	Sensitivity 0.868; precision 0.955; accuracy 0.859; Dice 0.862; mean IoU 0.758; ~0.15 s/image
Zhao 2022 [48]	Detection/localization	Training: 9524 images from 240 patients and 199 healthy subjects; test: 2312 images from 33 patients and 36 healthy subjects	Recognition of uterine fibroids	Sensitivity 94.21%; specificity 83.76%; accuracy 88.93%; F1 89.36%; AUC 0.96
Zhao 2023 [49]	Detection/localization	Training: 11,839 images from 323 cases; test: 431 cases from 2 hospitals	Real-time endometrial polyp detection	Lesion-based sensitivity 100% (internal) and 92.0% (external)
Chen 2023 [50]	Operative/technical support	56 patients with submucosal myomas	AI-augmented hysteroscopic myomectomy support	Operative time 41.32 ± 17.83 vs. 32.11 ± 11.86 min (*p* = 0.03); blood loss IQR reduced (*p* = 0.04)
Kitaya 2024 [51]	Diagnostic classification	244 women screened; 208 analyzed (78 with EMiP, 130 without)	Automatic detection of endometrial micropolyps	Sensitivity 93.6%; specificity 92.3%; accuracy 92.8%; precision 88.0%; F1 0.907; AUC 0.930
Li 2024 [52]	Prognostic/multimodal decision support	753 post-hysteroscopic adhesiolysis patients; 5014 revisited hysteroscopic images + EMR	1-year conception prediction; postoperative risk stratification; ART triage	AUC 0.967 (training), 0.936 (validation), 0.965 (test); analysis time 3.7 ± 0.8 s/patient; ART benefit OR 6.0 (95% CI 1.27–27.8) in mid-high risk group
Raimondo 2024 [53]	Diagnostic classification	1500 images from 266 patients	Classification and identification of intracavitary uterine lesions	Best classification with clinical factors: precision 80.11%, recall 80.11%, specificity 90.06%, F1 80.11%, accuracy 86.74%; identification F1 92.37%
Kürkçü 2024 [39]	Diagnostic classification	1482 images; internal test subset 88 images; expert check on 50 images	Endometrial abnormality/cancer-oriented object detection	YOLOv9c: mAP50 0.906, precision 0.894; expert-rated model accuracy 94–98% on 50 test images
Wang 2025 [54]	Diagnostic classification	49,646 training images from 1204 patients; 6228 images from 190 patients in 2 independent test datasets	AEH/EC vs. benign lesions	Internal: AUC 0.979, sensitivity 95.2%, specificity 91.3%, accuracy 94.1%; external: AUC 0.975, sensitivity 92.1%, specificity 100%, accuracy 93.3%
Mascarenhas 2025 [55]	Detection/localization	65 hysteroscopies; 33,239 frames; 37,512 annotated objects	Detection and classification of polyps with localization	Object level: recall 0.96, precision 0.95, mAP50 0.98; frame level: mean recall 0.75, precision 0.98, F1 0.82
Li 2025 [56]	Prognostic/multimodal decision support	555 cases; 4922 hysteroscopic images	Fertility assessment/pregnancy prediction after endometrial injury	AUCs 0.982, 0.992, 0.990; c-index 0.920–0.940; kappa vs. senior hysteroscopists 0.84–0.89
Givon 2026 [57]	Prognostic/multimodal decision support	345 procedures in 328 women	Prediction of incomplete hysteroscopic myomectomy	Incomplete resection 16.2%; AUROC 0.72; average precision 0.93

Abbreviations: AUB, abnormal uterine bleeding; AEH, atypical endometrial hyperplasia; ART, assisted reproductive technology; AUC, area under the curve; CAD, computer-aided diagnosis; c-index, concordance index; EC, endometrial cancer; EMiP, endometrial micropolyps; EMR, electronic medical record; F1, harmonic mean of precision and recall; FPS, frames per second; IoU, intersection over union; mAP50, mean average precision at an IoU threshold of 0.50; OR, odds ratio; ROI, region of interest; SF + GLDS, statistical features plus gray-level difference statistics; SVM, support vector machine; YOLO, You Only Look Once.

**Table 2:** Diagnostic classification and object detection studies.

Study	Population/Dataset	Input and Unit	Target Task	Reference Standard	Model	Validation	Main Outcomes
Vlachokosta 2013 [40]	77 women: 28 AUB, 10 EC, 39 without pathology	Hysteroscopic images; Image	3-class classification: normal vs. AUB vs. EC	Histopathology/clinical classification as reported	Texture + vessel features; ANN	Internal evaluation	Accuracy 91.2%; sens 93.6%; spec 83.8%
Neofytou 2015 [41]	516 ROIs from 52 subjects	Gamma-corrected hysteroscopic ROIs; ROI	Normal vs. abnormal endometrium/early EC-oriented CAD	Biopsy/histopathology as reported	Color-texture features (RGB/HSV/YCrCb); SVM and PNN	Internal evaluation	Best correct classification rate 81% (SF + GLDS with SVM)
Zhang 2021 [44]	1851 images from 454 patients; test set 250 images	Hysteroscopic images; Image	5-class lesion classification; benign vs. premalignant/malignant	Histopathology	VGGNet-16	Internal held-out test set	Overall accuracy 80.8%; benign vs. premalignant/malignant: accuracy 90.8%, sens 83.0%, spec 96.0%
Takahashi 2021 [45]	177 patients	Hysteroscopic images; Image/patient aggregation	EC-focused diagnosis from hysteroscopy	Clinical/pathological diagnosis as reported	Three DNN models + continuity analysis + combined model	Internal evaluation	Combined accuracy 90.29%; sens 91.66%; spec 89.36%
Kitaya 2024 [51]	244 women screened; 208 analyzed (78 with EMiP, 130 without)	Fluid hysteroscopic images; Image	Automatic detection of endometrial micropolyps	Expert EMiP assessment on F-HSC images; histologically confirmed CE cohort	CNN	Half-split training/test design	Sens 93.6%; spec 92.3%; accuracy 92.8%; precision 88.0%; F1 0.907; AUC 0.930
Raimondo 2024 [53]	1500 images from 266 patients	Hysteroscopic images ± clinical factors; Image	Classification and identification of intracavitary uterine lesions	Pathologically confirmed intrauterine lesions	Deep-learning model with/without clinical factors	Train/validation/test split	Best classification with clinical factors: precision 80.11%, recall 80.11%, spec 90.06%, F1 80.11%, accuracy 86.74%; identification F1 92.37%
Kürkçü 2024 [39]	1482 images; internal test subset 88 images; expert check on 50 images	Hysteroscopic images; Image/object	Endometrial abnormality/cancer-oriented object detection	Physician annotation; expert review on subset	YOLOv8/v9 variants with GUI prototype	Internal testing + expert verification subset	YOLOv9c: mAP50 0.906, precision 0.894; expert-rated model accuracy 94–98% on 50 test images
Wang 2025 [54]	49,646 training images from 1204 patients; 6228 images from 190 patients in 2 independent test datasets	Hysteroscopic images; Image/patient-level aggregation	AEH/EC vs. benign lesions	Pathology/expert-labeled image sets as reported	ECCADx; ResNet-50 with contrastive learning	Internal and external multicenter validation; comparison with endoscopists	Internal: AUC 0.979, sens 95.2%, spec 91.3%, accuracy 94.1%; external: AUC 0.975, sens 92.1%, spec 100%, accuracy 93.3%

Abbreviations: AEH, atypical endometrial hyperplasia; AUC, area under the curve; AUB, abnormal uterine bleeding; CAD, computer-aided diagnosis; CE, chronic endometritis; CNN, convolutional neural network; DNN, deep neural network; EC, endometrial cancer; ECCADx, endometrial cancer computer-aided diagnosis system; EMiP, endometrial micropolyps; F1, harmonic mean of precision and recall; GUI, graphical user interface; HSV, hue–saturation–value; mAP50, mean average precision at an IoU of 0.50; PNN, probabilistic neural network; RGB, red–green–blue; ROI, region of interest; SF + GLDS, statistical features plus gray-level difference statistics; SVM, support vector machine; YCrCb, luminance–chrominance color space; YOLO, You Only Look Once.

**Table 3:** Real-time lesion detection and localization studies.

Study	Population/Dataset	Input and Unit	Target Task	Reference Standard	Model	Validation	Main Outcomes
Zhao 2021 [46]	87 videos from 45 patients; additional test on 8 videos from 5 patients	Hysteroscopic videos/images; Image/video	Real-time hysteromyoma detection	Clinical labeling/manual annotation	YOLOv3 + DCGAN hybrid	Internal plus separate video test	Accuracy 91.73%; real-time speed 25 FPS
Zhao 2022 [48]	Training: 9524 images from 240 patients and 199 healthy subjects; test: 2312 images from 33 patients and 36 healthy subjects	Hysteroscopic images; Image	Recognition of uterine fibroids	Clinical labeling/manual annotation	CNN-Transformer hybrid network (UFCs)	Held-out test set	Sensitivity 94.21%; specificity 83.76%; accuracy 88.93%; F1 89.36%; AUC 0.96
Zhao 2023 [49]	Training: 11,839 images from 323 cases; test: 431 cases from 2 hospitals	Hysteroscopic videos/images; Lesion/image/video	Real-time endometrial polyp detection	Clinical labeling/annotation	Improved YOLOX + group normalization + video adjacent-frame association algorithm	Internal and external test sets; real-time video evaluation	Lesion-based sensitivity 100% (internal) and 92.0% (external)
Mascarenhas 2025 [55]	65 hysteroscopies; 33,239 frames; 37,512 annotated objects	Full-length hysteroscopy videos/extracted frames; Object/frame	Detection and classification of polyps with localization	Histologically confirmed polyps; bounding-box annotation	YOLOv1-based object detection model	70/20/10 train/validation/test split	Object level: recall 0.96, precision 0.95, mAP50 0.98; frame level: mean recall 0.75, precision 0.98, F1 0.82

Abbreviations: AUC, area under the curve; CNN, convolutional neural network; UFCs, uterine fibroids classification system; DCGAN, deep convolutional generative adversarial network; F1, harmonic mean of precision and recall; FPS, frames per second; YOLO, You Only Look Once.

**Table 4:** Segmentation and visual-field support studies.

Study	Population/Dataset	Input and Unit	Target Task	Reference Standard	Model	Validation	Main Outcomes
Török 2018 [42]	13 surgical cases; 4688 training images and 1600 test images	Operative hysteroscopic video frames; Image/pixel	Segmentation of normal myometrium vs. myoma plane	Manual annotations	FCNN	Internal test set	Pixel-wise segmentation accuracy 86.19%
Burai 2018 [43]	28 videos; 1425 subimages	Hysteroscopic video frames; Image/pixel	Uterine wall segmentation under occluding conditions	Expert annotations	Ensemble FCNNs	Internal experiments	Segmentation accuracy 91.56%; Dice 0.9156; Jaccard 0.8443
Wang 2022 [47]	12 videos from 12 patients; 1385 clear images	Operative hysteroscopic images; Image/pixel/bubble instance	Bubble segmentation and size distribution	Manual labels	Edge-aware encoder–decoder network + marker-controlled watershed	Internal evaluation	Sensitivity 0.868; precision 0.955; accuracy 0.859; Dice 0.862; mean IoU 0.758; ~0.15 s/image

Abbreviations: Dice, Dice similarity coefficient; FCNN, fully convolutional neural network; IoU, intersection over union; Jaccard, Jaccard index; s/image, seconds per image.

**Table 5:** Operative guidance study.

Study	Population/Dataset	Input and Unit	Target Task	Reference Standard	Model	Validation	Main Outcomes
Chen 2023 [50]	56 patients with submucosal myomas	MRI + AI-assisted operative planning/segmentation context; Patient	AI-augmented hysteroscopic myomectomy support	Operative outcomes	Deep-learning segmentation/localization suite	Two-arm clinical comparison	Operative time 41.32 ± 17.83 vs. 32.11 ± 11.86 min (*p* = 0.03); blood loss IQR reduced (*p* = 0.04)

Abbreviations: AI, artificial intelligence; MRI, magnetic resonance imaging; min, minutes.

**Table 6:** Prognostic, multimodal, and hysteroscopy-related decision-support studies.

Study	Population/Dataset	Input and Unit	Target Task	Reference Standard	Model	Validation	Main Outcomes
Li 2024 [52]	753 post-hysteroscopic adhesiolysis patients; 5014 revisited hysteroscopic images + EMR	Hysteroscopic images + EMR; Patient	1-year conception prediction; postoperative risk stratification; ART triage	Observed reproductive outcomes	MobilenetV3 + XGBoost multimodal ensemble	Training, validation, test datasets	AUC 0.967 (training), 0.936 (validation), 0.965 (test); analysis time 3.7 ± 0.8 s/patient; ART benefit OR 6.0 (95% CI 1.27–27.8) in mid-high risk group
Li 2025 [56]	555 cases; 4922 hysteroscopic images	Hysteroscopic images; Patient	Fertility assessment/pregnancy prediction after endometrial injury	Observed reproductive outcomes	Proportional hazard CNN system	Three randomly assigned datasets	AUCs 0.982, 0.992, 0.990; c-index 0.920–0.940; kappa vs. senior hysteroscopists 0.84–0.89
Givon 2026 [57]	345 procedures in 328 women	Preoperative clinical and imaging variables including ultrasound and diagnostic hysteroscopy findings; Procedure/patient	Prediction of incomplete hysteroscopic myomectomy	Documented residual submucosal myoma at end of surgery	CatBoost classifier; comparison with logistic regression	Stratified 5-fold patient-level cross-validation	Incomplete resection 16.2%; AUROC 0.72; average precision 0.93

Abbreviations: ART, assisted reproductive technology; AUC, area under the curve; AUROC, area under the receiver operating characteristic curve; CatBoost, categorical boosting classifier; c-index, concordance index; CNN, convolutional neural network; EMR, electronic medical record; OR, odds ratio; XGBoost, extreme gradient boosting.

## Data Availability

No new data were created or analyzed in this study. Data sharing is not applicable to this article.

## Supplementary Materials

The following supporting information can be downloaded at: https://www.mdpi.com/article/10.3390/diagnostics16121899/s1, PRISMA 2020 Checklist.

## Appendix A

**Table A1:** Practical definitions of key technical terms used in this review.

Term	Practical Meaning in This Review
Artificial intelligence (AI)	Computational systems that perform tasks usually requiring human pattern recognition or decision-making.
Machine learning (ML)	AI approach in which models learn patterns from data instead of manually written diagnostic rules.
Deep learning (DL)	ML approach based on multilayer neural networks, particularly useful for image analysis.
Convolutional neural network (CNN)	Deep-learning model designed to analyze images by detecting visual patterns such as edges, texture, color, shape, and lesion morphology.
Computer-aided diagnosis (CAD)	Clinical use of AI/ML/DL outputs to support detection, classification, or diagnostic decision-making.
Object detection	Image-analysis task in which the model identifies and localizes a target lesion, usually by drawing a bounding box around it.
Segmentation	Image-analysis task in which the model outlines the exact pixels belonging to a structure or lesion, such as a polyp, myoma plane, uterine wall, or bubble.
Intersection over union (IoU)	Measure of overlap between the model-detected region and the reference annotation; higher values indicate better localization.
Mean average precision (mAP)	Object-detection metric that combines how often the model finds the target and how precisely it localizes it.
Data leakage	Methodological error in which related images, frames, or patients appear in both training and test data, leading to overly optimistic performance.
Domain shift	Loss of model performance when the system is used in settings different from the training data, such as another center, camera system, operator, or acquisition protocol.
Calibration	Agreement between predicted probability and observed risk; for example, whether a predicted 20% risk corresponds to an actual event rate near 20%.

**Table A2:** Risk of bias and quality assessment of included studies.

Study	Study Type	Instrument *	Overall Judgment	Principal Concerns
Vlachokosta 2013 [40]	Diagnostic classification	QUADAS-2	High/unclear risk	Single-center, selected images, no external validation, limited reporting of split strategy
Neofytou 2015 [41]	Diagnostic classification	QUADAS-2	High/unclear risk	ROI-level analysis, small sample, no external validation, developmental study
Török 2018 [42]	Operative/technical support	Technical quality domains	Moderate–high concerns	Very small case base, no external validation, manual annotation reference
Burai 2018 [43]	Operative/technical support	Technical quality domains	Moderate–high concerns	Conference paper, limited reporting, one developmental dataset, no external validation
Zhang 2021 [44]	Diagnostic classification	QUADAS-2	High risk	Single center, internal validation only, small fixed test set, heavy augmentation
Takahashi 2021 [45]	Diagnostic classification	QUADAS-2	Some concerns to high risk	Small malignant subgroup, no external validation, retrospective single-center design
Zhao 2021 [46]	Detection/localization	Technical quality domains	Moderate–high concerns	Conference format, limited reporting, small testing basis, no external validation
Wang 2022 [47]	Operative/technical support	Technical quality domains	Moderate concerns	Small dataset, no external validation, technical rather than patient-level clinical endpoint
Zhao 2022 [48]	Detection/localization	Technical quality domains	Moderate–high concerns	Conference paper, limited reporting, no external validation
Zhao 2023 [49]	Detection/localization	QUADAS-2	Moderate concerns	Good multicenter testing, but training from one hospital and limited calibration/clinical deployment data
Chen 2023 [50]	Operative/technical support	RoB 2	Some concerns	Small sample; reporting of allocation/concealment limited in accessible full text
Kitaya 2024 [51]	Diagnostic classification	QUADAS-2	Moderate–high concerns	Single center, internal split only, selection from screened cohort, no external validation
Li 2024 [52]	Prognostic/multimodal decision support	PROBAST	High risk	Internal split within one platform/cohort line, no truly external validation cohort
Raimondo 2024 [53]	Diagnostic classification	QUADAS-2	High risk	Single center, retrospective, no external validation, moderate case mix limitations
Kürkçü 2024 [39]	Diagnostic classification	Technical quality domains	High/unclear concerns	Conference format, limited test set, partial reporting of validation and reference standard
Wang 2025 [54]	Diagnostic classification	QUADAS-2	Moderate concerns	Retrospective AI development study, but large multicenter data and external testing strengthen credibility
Mascarenhas 2025 [55]	Detection/localization	QUADAS-2	High/unclear concerns	Multicenter histology-confirmed dataset, but object-level split risks same-procedure leakage; no prospective real-time validation.
Li 2025 [56]	Prognostic/multimodal decision support	PROBAST	High risk	Internal random-split validation within same cohort line; no independent external validation
Givon 2026 [57]	Prognostic/multimodal decision support	PROBAST	High risk	No external validation; model developed and tested within same center/cohort

* QUADAS-2 was used for diagnostic studies, PROBAST for prognostic/predictive model studies, and structured technical quality domains for studies whose endpoints are primarily segmentation, visualization, or engineering feasibility. Abbreviations: PROBAST, Prediction model Risk Of Bias Assessment Tool; QUADAS-2, Quality Assessment of Diagnostic Accuracy Studies 2; RoB 2, revised Cochrane risk-of-bias tool for randomized trials.
